# Gut Microbiota‐Derived Acetate Ameliorates Endometriosis via JAK1/STAT3‐Mediated M1 Macrophage Polarisation

**DOI:** 10.1111/1751-7915.70202

**Published:** 2025-07-30

**Authors:** Yunyun Xu, Yichen Zhu, Xiaoyun Wu, Wan Peng, Yanying Zhong, Yujie Cai, Wenjing Chen, Lu Liu, BuZhen Tan, Tingtao Chen

**Affiliations:** ^1^ Department of Obstetrics and Gynecology, The Second Affiliated Hospital, Jiangxi Medical College Nanchang University Nanchang Jiangxi China; ^2^ School of Pharmacy, Jiangxi Medical College Nanchang University Nanchang China; ^3^ Jiangxi Province Key Laboratory of Bioengineering Drugs, School of Pharmacy, Institute of Translational Medicine, Jiangxi Medical College Nanchang University Nanchang Jiangxi China

**Keywords:** acetate, endometriosis, faecal microbiota transplantation, gut microbiota, JAK–STAT signalling, macrophage polarisation, short‐chain fatty acids (SCFAs)

## Abstract

Endometriosis (EMs) is a common inflammatory disorder in women of reproductive age, severely impacting patients' quality of life and fertility. Current hormonal therapies offer limited efficacy, and surgical interventions often fail to prevent recurrence. Recent studies suggest a close association between gut microbiota and the pathophysiology of EMs, though the precise mechanisms remain unclear. To investigate the influence of gut microbiota on EMs, this study established an EMs mouse model and performed faecal microbiota transplantation (FMT) using samples from healthy donors (AH group) and EMs patients (AE group) into the model mice. Results demonstrated that compared to the model group (M group), FMT from healthy donors (AH group) significantly reduced ectopic lesion volume (658.3 ± 116.1 vs. 167.2 ± 112.8 mm^3^, *p* < 0.01) and weight (0.7420 ± 0.1233 vs. 0.1885 ± 0.1239 mg, *p* < 0.01). Conversely, FMT from EMs patients exacerbated disease progression. Mechanistic studies revealed that healthy donor FMT attenuated EMs by remodelling the gut microbial composition (enhancing α‐diversity and *Lactobacillus* abundance while suppressing Bacteroidetes), significantly elevating acetate levels in faeces and ectopic lesions, activating the JAK1/STAT3 signalling pathway within lesions, and thereby driving macrophage polarisation toward the M1 phenotype (by increased iNOS/CD86 expression and decreased Arg1/CD206 expression). Simultaneously, healthy donor FMT enhanced intestinal barrier integrity by upregulating tight junction proteins (ZO‐1, Occludin, Claudin‐1/5) and reducing levels of intestinal permeability markers (DAO, IFABP). In contrast, AE group FMT disrupted gut microbial ecology, reduced acetate production, failed to activate the JAK1/STAT3 pathway, promoted M2 macrophage polarisation and impaired intestinal barrier function. Collectively, this study elucidates for the first time that acetate, as a key gut microbiota metabolite, exerts anti‐EMs effects by activating the JAK1/STAT3 signalling pathway to drive macrophage reprogramming toward the M1 phenotype, thereby positioning gut microbiota reconstruction as a novel therapeutic strategy for endometriosis.

## Graphical abstract

This study first reveals acetate as a key gut microbiota metabolite to ameliorate endometriosis (EMs) via JAK1/STAT3‐mediated M1 macrophage polarisation, confirming healthy donor faecal microbiota transplantation (FMT) improves EMs by reshaping microbiota, enhancing acetate, strengthening intestinal barriers and providing a new therapeutic paradigm.

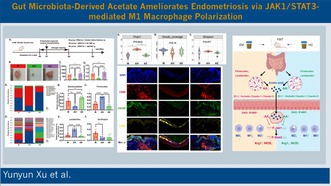

## 1 Introduction

Endometriosis (EMs) is an oestrogen‐dependent chronic inflammatory disorder affecting approximately 198 million women globally. It is characterised by chronic pelvic pain, ovarian endometriotic cysts and infertility, significantly impairing patients' quality of life (Taylor et al. 2021). Due to its insidious progression and poorly understood pathogenesis, EMs is often diagnosed late (Agarwal et al. 2019). Current treatments, including surgical lesion removal, nonsteroidal anti‐inflammatory drugs (NSAIDs) and hormonal therapy, are suboptimal, with a 50% recurrence rate within 5 years (Horne and Missmer 2022). This imposes substantial physical, emotional and economic burdens on patients and healthcare systems (Saunders and Horne 2021). Therefore, elucidating the molecular mechanisms underlying EMs pathogenesis is critical for developing more effective therapeutic strategies.

The pathogenesis of EMs is complex, with the immune and inflammation theories being one of the widely accepted hypotheses (Wang et al. 2020). Macrophages, as the main immune cells in endometriotic lesions, play a key role in the growth, development, angiogenesis, innervation and pain symptoms of the lesions (Ramírez‐Pavez et al. 2021). However, the specific mechanisms driving the increase in macrophage numbers and their functional roles in endometriosis have not been fully elucidated, although their abnormal phenotypes and functional disorders significantly impact disease progression. Macrophages exhibit two distinct polarisation states: the classically activated M1 phenotype, identified by the expression of CD40, CD80, CD86 and HLADR, and the alternatively activated M2 phenotype, distinguished by the expression of CD163, CD206 and CD204 (Li et al. 2021). In endometriosis, M1 macrophages attract immune cells to ectopic lesions by releasing pro‐inflammatory factors (such as IL‐1β and TNF‐α) in the early stages, triggering inflammatory responses and inhibiting lesion growth. In contrast, as the disease progresses, the number of M2 macrophages increases, secreting vascular endothelial growth factor (VEGF) and IL‐10, creating an anti‐inflammatory environment that promotes the adhesion, proliferation and angiogenesis of ectopic endometrium, thereby driving lesion growth (Ramírez‐Pavez et al. 2021; Yan et al. 2023). Studies have shown that regulating macrophage polarisation may be a key therapeutic strategy for managing endometriosis (Yuan et al. 2023; Miller et al. 2020; Kobayashi and Imanaka 2022). Yan Shumin found that *Escherichia coli* inhibits the migration and proliferation of co‐cultured endometrial cells in vitro through IL‐1, prevents lesion growth in vivo, induces macrophage polarisation toward the M1 phenotype, and thereby inhibits the progression of endometriosis (Yan et al. 2023). In addition, Li Qiuju's research indicates that nanovesicles derived from M1 macrophages (M1NVs) can not only directly hinder the progression of endometriosis but also promote the repolarisation of macrophages from the M2 phenotype to the M1 phenotype, indirectly inhibiting the migration and invasion of endometrial stem cells in patients (Li et al. 2021). Therefore, M2 macrophages are believed to promote the progression of endometriosis, while M1 macrophages play an inhibitory role, and treatment strategies aimed at promoting M1 polarisation are crucial for improving the outcomes of EMs.

The gut microbiota is intricately linked to various intestinal and extraintestinal disorders, influencing the host's gut environment, barrier function, and secretory and immune functions through the production and modification of metabolites such as short‐chain fatty acids (SCFAs), bile acids (BAs) and lipopolysaccharides (LPS) (Mann et al. 2024; Winston and Theriot 2020; Liu et al. 2023; He, Xiong, et al. 2024). Patients with EMs exhibit significant gut microbiota dysbiosis. Svensson et al. found that 58 bacterial genera were observed in the control and EMs patient groups, with the control group having higher α and β diversity and significant differences in the abundance of 12 bacterial genera, including *Bacteroides* and *Clostridium* (Svensson et al. 2021). Wei et al. discovered that gut microbiota imbalance can lead to macrophage dysfunction, affecting inflammation and neurovascular growth in EMs, and elevated oestrogen levels within lesions increase vascular permeability, promoting macrophage infiltration (Wei et al. 2023). Gut microbiota dysbiosis can disrupt immune balance, and acetate, an important metabolite of the gut microbiota, can promote macrophage polarisation toward anti‐inflammatory or pro‐inflammatory phenotypes through various means such as altering intracellular metabolic status, regulating signalling pathways and affecting epigenetic modifications, thereby influencing the progression of various diseases (Machado et al. 2022; Ma et al. 2024; Erny et al. 2021). In hepatocellular carcinoma (HCC), acetate produced by *Bacteroides thetaiotaomicron* can promote M1 polarisation of macrophages, enhancing the function of cytotoxic CD8+ T cells, thereby inhibiting tumour growth (Ma et al. 2024). Additionally, Li et al. demonstrated that in nonalcoholic fatty liver disease (NAFLD), acetate dose‐dependently regulates macrophage inflammatory responses, with low doses promoting inflammation and M1 polarisation, and high doses inhibiting; it also changes intracellular acetate concentration, affects metabolism and inflammation through the AMPK signalling pathway, and bidirectionally regulates macrophage activity, thereby influencing NAFLD progression (Li et al. 2023). However, the role of acetate in the immune disease EMs has not been fully explored, and its direct in vivo effects remain largely unknown.

Faecal microbiota transplantation (FMT) is a novel therapeutic strategy that introduces gut microbiota from healthy donors into the intestinal tract of recipients to reestablish microbial equilibrium, offering a potential treatment for disorders linked to microbial imbalance (Benech and Sokol 2020). The mechanisms of FMT include modulating the immune system, restoring microbial balance and regulating metabolism (Benech and Sokol 2020; O'Leary 2021; Lavelle and Sokol 2022). However, it is currently unclear whether FMT can effectively improve symptoms of EMs and whether this process involves the regulation of macrophage polarisation and its underlying mechanisms, which remain a research gap.

This study successfully established a mouse model of endometriosis, aiming to investigate the effects of FMT on the disease phenotype of endometriosis and the gut microbiota of mice, and to further explore the role and underlying mechanisms of macrophage polarisation in this process. The results showed that FMT significantly improved endometriosis by regulating gut microbiota imbalance, enhancing gut barrier function and promoting the generation of acetate to drive macrophage polarisation toward the M1 phenotype. In summary, this study provides the first experimental evidence that FMT derived from healthy donors can ameliorate endometriosis. Furthermore, it identifies acetate as a potential key metabolite regulating macrophage polarisation in this context. These findings offer novel perspectives and scientific rationale for developing clinical therapeutic strategies for this disease, demonstrating significant clinical implications for improving patient outcomes.

## 2 Materials and Methods

### 2.1 Subjects Enrollment and Ethical Approval

Stool specimens were collected from a clinical cohort recruited at the Second Affiliated Hospital of Nanchang University, China, between 3 January 2024 and 30 June 2024. The study protocol was approved by the Biomedical Research Ethics Committee of the hospital (Approval No. [2024]01), and written informed consent was obtained from all participants after a detailed explanation of the study objectives and procedures.

The cohort included four endometriosis patients (diagnosed by postoperative histopathology, including 2 with r‐ASRM stage 1–2 and 2 with stage 3–4) and four healthy female controls. Inclusion criteria comprised: (1) age 18–45 years with a regular menstrual cycle (28 ± 7 days); (2) diagnosis confirmed by postoperative imaging and pathology; (3) no antibiotic, glucocorticoid or probiotic use in the past 3 months, and no hormone therapy in the past 6 months; (4) normal white blood cell count and neutrophil ratio on routine blood tests. Exclusion criteria were: (1) age under 18 or over 45, or postmenopausal status; (2) any medication use in the 3 months prior to the study (excluding gonadotropin‐releasing hormone analogs), including oral contraceptives, progesterone, endometriosis treatments or probiotics; (3) pregnancy or breastfeeding; (4) history of surgery, autoimmune diseases, systemic acute inflammation or intestinal infections.

### 2.2 Gut Microbiota Depletion and Faecal Microbiota Transplantation (FMT)

Prior to transplantation, mice were pre‐treated with antibiotics by daily gavage of a mixture containing vancomycin (100 mg/kg), neomycin sulfate (200 mg/kg), metronidazole (200 mg/kg) and ampicillin (200 mg/kg) for 7 consecutive days to eliminate their indigenous gut microbiota. Fresh faecal samples from EMs patients and healthy women were immediately mixed with five volumes of sterile saline, homogenised, filtered through 0.45 μm cell strainers, and centrifuged (1000×*g*, 4°C, 5 min). The collected supernatant was suspended in 30% (v/v) glycerol and stored at −80°C for later use. For FMT, frozen suspensions were thawed, centrifuged, resuspended in gelatin saline and administered via oral gavage at 100 μL per mouse, twice weekly for 4 weeks (Hu et al. 2023).

### 2.3 Animals and Endometriosis Model Establishment

A model of endometriosis in mice was established through the allotransplantation of uterine tissue, following the methodology described by Chadchan et al. (2023). The experimental design included nine female BALB/c donor mice, aged 8 weeks and weighing 18–22 g. These mice were given a daily oral gavage of 56 μg/kg/day of 17β‐estradiol (Huachu, Xinxiang, China) for 3 days prior to euthanasia. After the donor mice were euthanised, the uterine horns were surgically removed and immediately placed in sterile PBS at 37°C. Subsequently, the uterine horns were longitudinally incised and halved using micro‐scissors, providing four samples per uterus (approximately 2 × 2 mm in size) for transplantation into the peritoneal cavities of recipient mice. Tissue fragment dimensions were standardised under a surgical microscope using a calibrated ocular micrometre, consistent with established protocols for murine endometriosis modelling (Burns et al. 2012; Grümmer 2006). All experimental groups (M/AH/AE) utilised endometrial tissues from the same cohort of donor mice, with fragment preparation and suturing performed by a single operator.

Eighteen female BALB/c recipient mice, age‐ and weight‐matched to the donors, were anaesthetised using isoflurane and placed on mechanical ventilation. A midline abdominal incision was performed, and two sections of the uterine horn were attached to the parietal peritoneum with 7/0 polypropylene sutures (Prolen, Ethicon, Somerville, NJ), one on each side. To standardise neovascularisation at the implantation site, all tissue samples were sutured to the same location on the abdominal wall. The incisions were then closed using 6/0 nylon sutures. Beginning 3 days post‐transplantation, all recipient mice were administered 56 μg/kg/day of 17β‐estradiol (Huachu, Xinxiang, China) via oral gavage as part of their postoperative regimen.

To investigate the role of gut microbiota in endometriosis, mice subjected to modelling were randomly divided into three groups: (1) Model (M) group (*n* = 6): Received an equal volume of saline via gavage; (2) AH group (faecal microbiota from healthy females): Following modelling and a 1‐week antibiotic regimen, mice were orally given 100 μL of faecal suspension from healthy donors twice weekly for 4 weeks, *n* = 6; (3) AE group (faecal microbiota from EMs patients): After modelling and a 1‐week antibiotic treatment, mice were orally administered 100 μL of faecal suspension from EMs patients twice weekly for 4 weeks, *n* = 6.

All procedures related to animal experiments were approved by the Ethics Committee for Animal Welfare of Nanchang University (Approval No: NCULAE‐20221031116) and conducted according to the approved guidelines.

### 2.4 Metabolomic Analysis

Gas chromatography–mass spectrometry (GC–MS) was employed to characterise the metabolites in the samples. A 20 mg sample was mixed with 1 mL of 0.5% phosphoric acid and steel balls, followed by processing through grinding, vortexing and sonication. After centrifugation, 0.1 mL of the supernatant was combined with 0.5 mL of methyl tert‐butyl ether (MTBE), subjected to vortexing, sonication and another centrifugation step. The GC–MS analysis was performed using an Agilent 7890B gas chromatograph and a 7000D mass spectrometer equipped with a DB‐5MS column.

### 2.5 Haematoxylin–Eosin (H&E) Staining

Endometriosis lesions and intestinal tissue samples were collected and immersed in 4% paraformaldehyde for fixation. Following standard histological processing including alcohol dehydration, the specimens were encased in paraffin wax blocks. Thin longitudinal sections of 5 μm thickness were prepared using a Leica CM1850 microtome (Leica Microsystems, Germany). Tissue sections underwent routine H&E staining before being mounted onto glass slides for microscopic evaluation. Histomorphological analysis was conducted using a Nikon Eclipse 80i optical microscope (Nikon Instruments, Japan) integrated with NIS‐Elements 3.2 imaging software for detailed cellular examination.

### 2.6 Immunohistochemistry

Paraffin‐embedded tissue sections underwent dewaxing with xylene followed by sequential rehydration in ethanol solutions at decreasing concentrations. Heat‐mediated antigen retrieval was performed using sodium citrate buffer (Vector Laboratories, USA) through microwave irradiation. Sections were then treated with PBS containing 2.5% normal goat serum (Vector Laboratories) as blocking solution under dark humidified conditions for 60 min. Following PBS rinsing, primary antibody incubation was conducted at 4°C for 12–16 h. After triple PBS washes, fluorescent labeling was achieved using Alexa Fluor 488‐linked secondary antibodies (1:500 dilution; Thermo Fisher) incubated for 60 min at ambient temperature. Processed sections were coverslipped with DAPI‐embedded ProLong Gold mounting medium (Thermo Fisher) for nuclear counterstaining and fluorescence preservation.

### 2.7 Immunofluorescence Staining

Initially, paraffin‐embedded sections were generated. After deparaffinisation and hydration, these sections underwent cleansing with PBS and were subjected to heat‐mediated antigen retrieval. Following a 30‐min occlusion with 3% BSA, the sections were exposed to primary antibodies targeting CD86 (Cat No. 83213‐1‐RR, sourced from Proteintech, Wuhan, China), CD206 (Cat No. 18704‐1‐AP, Proteintech, Wuhan, China) and CD68 (Cat No. 28058‐1‐AP, Proteintech, Wuhan, China) overnight at a temperature of 4°C. Post PBS rinsing, the sections were incubated with a secondary antibody (SA00013‐2, Proteintech, USA) for a duration of 2 h at ambient temperature, shielded from light. This was succeeded by nuclear staining with DAPI (#AWC0293a, Abiowell, China). Ultimately, the sections were preserved with a 10% glycerol solution and visualised utilising a fluorescence microscope.

### 2.8 Biochemical Analysis

Serum diamine oxidase (DAO) and intestinal fatty acid binding Protein (IFABP) were determined using ELISA kits (MEIMIAN, China) according to the manufacturer's instructions.

### 2.9 Quantitative Real Time (RT)—PCR

Total RNA was isolated from endometriotic tissue samples using Trizol reagent (Invitrogen), followed by quantification of RNA purity and concentration with a NanoDrop 2000 spectrophotometer (Thermo Scientific, USA). Reverse transcription was performed to generate cDNA from RNA templates, utilising a commercial kit (Invitrogen) as per the provided guidelines. The cDNA synthesis protocol involved sequential incubation at 16°C (30 min), 42°C (30 min), and 85°C (5 min), with final storage at 4°C. Target gene HOTAIR was amplified and quantified through TaqMan probe‐based real‐time PCR, employing a thermal cycling profile of initial denaturation at 95°C for 10 min, followed by 40 cycles of 95°C for 15 s and 60°C for 1 min. Primer sequences specific to HOTAIR are detailed in Table 1. Relative quantification of gene expression was determined by the 2^−ΔΔCt^ method, with β‐actin serving as the internal reference gene for normalisation purposes, and the primer sequences are shown in Table 1.

**TABLE 1:** Primer sequences for quantitative RT‐qPCR.

Primers	Forward (5′–3′)	Reverse (5′–3′)
ARG‐1	CTGAGAGATTCAAGGCAAGAGG	GAACGCGCTATCTTACCCCAG
CD206	CTCTGTTCAGCTATTGGACGC	TGGCACTCCCAAACATAATTTGA
iNOS	CTCTTCGACGACCCAGAAAAC	CAAGGCCATGAAGTGAGGCTT
CD86	TCAATGGGACTGCATATCTGCC	GCCAAAATACTACCAGCTCACT
JAK1	AGTGCAGTATCTCTCCTCTCTG	GATTCGGTTCGGAGCGTACC
STAT3	CACCTTGGATTGAGAGTCAAGAC	AGGAATCGGCTATATTGCTGGT
AMPK	TCTGAGGGGCACCAAGAAAC	GTGGGTGTTGACGGAGAAGAG
ERK1	TCCGCCATGAGAATGTTATAGGC	GGTGGTGTTGATAAGCAGATTGG
ERK2	TCAGATGAATTTTCGTTGGCAGA	AGCTTTTGTATTGGTCACAGCA
ZO‐1	CTCCAGAGCACCGAGAGCTA	GGCGTTTGCGAAGTTCTTCAT
Occludin	TGAAAGTCCACCTCCTTACAGA	CCGGATAAAAAGAGTACGCTGG
Claudin‐1	CTTGGATTCTTCGGTTTGGTTGG	CTGCCGATGAAAGCTGACAC
Claudin‐5	GCAAGGTGTATGAATCTGTGCT	GTCAAGGTAACAAAGAGTGCCA
β‐Actin	GTGACGTTGACATCCGTAAAGA	GCCGGACTCATCGTACTCC

### 2.10 DNA Extraction and 16S rDNA Gene Sequencing

Following the procurement of faecal specimens, these were preserved at a temperature of −80°C to facilitate the subsequent DNA extraction procedure. This operation was conducted utilising genomic DNA extraction kits, specifically models M5636‐02 supplied by Omega in the United States and D6356‐03 by Magen in China, adhering strictly to the guidelines furnished by the respective manufacturers. The quantification and assessment of the DNA's purity were accomplished via the NanoDrop system, a product of Thermo Fisher Scientific based in the USA. For the amplification of the V4 segment of the 16S ribosomal DNA (rDNA) gene, a pair of barcoded primers with sequences 5′‐AYTGGGYDTAAAGNG‐3′ and 5′‐TACNVGGGTATCTAATCC‐3′ were employed. The ensuing sequencing was performed on the Illumina Novaseq platform.

### 2.11 Data Analysis

Data processing and statistical evaluation were conducted using GraphPad Prism 8.0 software for visualisation and preliminary analysis, complemented by SPSS 17.0 for advanced statistical computations. Continuous variables underwent distribution pattern assessment via the Shapiro–Wilk algorithm to determine normality compliance. Quantities conforming to Gaussian distribution were expressed as mean ± SD values, whereas intergroup differences were analysed using single‐factor ANOVA with subsequent application of Tukey's multiple comparison adjustment. For non‐parametric datasets, the Kruskal–Wallis rank‐sum test was implemented as an omnibus test. When significant differences were detected (*p* < 0.05), post hoc pairwise comparisons were performed using Dunn's test with Bonferroni correction. Results were expressed as median values with interquartile ranges (IQR). Discrete variables were characterised by frequency counts and percentages, with group differences assessed using either the chi‐square test or Fisher's exact probability test for limited sample sizes. A probability threshold of *p* < 0.05 was established as the criterion for statistical significance (Lin et al. 2024; He, Lin, et al. 2024; He, Li, et al. 2022; He, Wang, et al. 2022).

## 3 Results

### 3.1 Characteristics of the Included Clinical Study Population

This study included four patients with endometriosis and four healthy women. The revised American Society for Reproductive Medicine (r‐ASRM) scoring system was used to assess the severity of EMs, with two patients classified as stage 3–4 and two patients as stage 1–2. All EMs patients were confirmed by pathological examination post‐surgery. Detailed demographic and clinical characteristics of the participants are summarised in Table 2. There were no significant differences between the EMs and control groups in terms of age (36.00 ± 4.378 vs. 37.00 ± 5.050, *p* = 0.8860) and body mass index (BMI, 21.71 ± 1.414 vs. 20.05 ± 0.4174, *p* = 0.3038). However, the abortion rate was higher in the EMs group compared to the control group (3.750 ± 0.4787 vs. 1.750 ± 0.4787, *p* = 0.0255). In relevant tests, anti—Müllerian hormone (AMH), an indicator of ovarian reserve, there was no significant difference between the two groups (4.000 ± 0.6494 vs. 3.125 ± 0.6700, *p* = 0.9601). Cancer antigen 125 (CA125) levels were significantly higher in the EM group (32.00 ± 4.021 vs. 218.3 ± 38.71; *p* = 0.003).

**TABLE 2:** Characteristics of the included clinical study population.

Variable	Control group (*n* = 4)	Endometriosis group (*n* = 4)	*p*
Age (years)	36.00 ± 4.378	37.00 ± 5.050	0.8860
BMI	21.71 ± 1.414	20.05 ± 0.4174	0.3038
Abortions (number)	1.750 ± 0.4787	3.750 ± 0.4787	0.0255
AMH (ng/mL)	4.000 ± 0.6494	3.125 ± 0.6700	0.9601
CA125 (U/mL)	32.00 ± 4.021	218.3 ± 38.71	0.0030
r‐ASRM score	Stage 1/2	2	
	Stage 3/4	2	

### 3.2 The Gut Microbiota Plays a Crucial Role in the Pathogenesis and Progression of EMs

**FIGURE 1:**
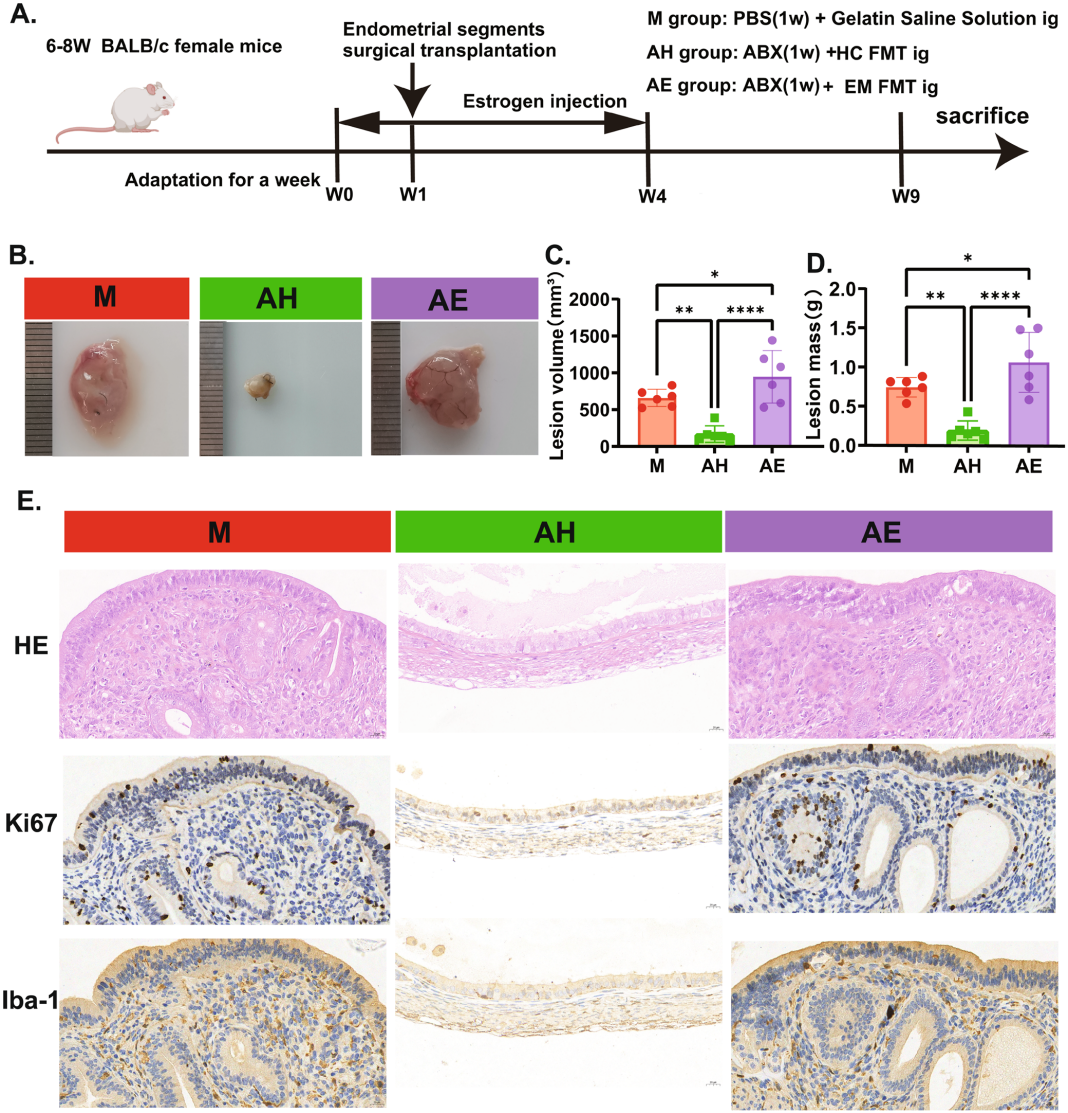
Effects of FMT on endometriosis progression in mice. (A) Experimental design: endometriosis was modelled in 6–8‐week‐old female BALB/c mice by surgically transplanting endometrial fragments and administering oestrogen injections. Mice were intragastrically treated with Abx for 1 week, followed by experimental intervention with 200 μL faecal suspension via oral gavage every 2 days for 4 weeks. (B) Representative images of endometriotic lesions in each group. (C) Volume of lesions. (D) Weight of lesions. (E) Representative images of HE stains and immunohistochemical staining for Ki67 and Iba‐1 in lesions from each group. Scale bar: 20 μm. AE, FMT from endometriosis patients; AH, FMT from healthy women; M, model group. Data are presented as mean ± standard deviation. *p < 0.05, **p < 0.01, ***p < 0.001, ****p < 0.0001.

To investigate the modulatory role of gut microbiota in EMs progression, BALB/c mice were administered a broad‐spectrum antibiotic mixture (Abx) for 1 week to eliminate resident intestinal microorganisms. FMT was then performed using donor samples from healthy individuals (AH group) or EMs patients (AE group) through gastric intubation (Figure 1A). Post 4‐week intervention, AH recipients exhibited diminished ectopic lesions compared to model controls (M group), whereas AE recipients displayed lesion expansion (Figure 1B). Quantitative analysis revealed substantial reductions in AH group's lesion volume (658.3 ± 116.1 vs. 167.2 ± 112.8 vs. 944.3 ± 355.9, *p* < 0.0001, Figure 1C) and mass (0.7420 ± 0.1233 vs. 0.1885 ± 0.1239 vs. 1.059 ± 0.3830, *p* < 0.0001, Figure 1D) relative to M and AE groups, demonstrating healthy microbiota's inhibitory effects versus EM‐derived microbiota's disease‐promoting capacity. Mechanistic investigations commenced with histopathological evaluation through H&E staining, confirming model mice displayed characteristic EMs architecture with hyperplastic epithelial layers and glandular proliferation. While AE and M groups maintained comparable epithelial thickness and stromal organisation, AH specimens showed attenuated epithelial/stromal dimensions and decreased glandular density (Figure 1E). Subsequent analysis of epithelial proliferation using Ki‐67 immunostaining revealed significantly fewer positive cells in AH group versus model controls, contrasting with AE group's elevated proliferative activity (Figure 1E). Macrophage infiltrate density, a critical microenvironment modulator through pro‐inflammatory cytokine secretion and angiogenesis regulation, was quantified via Iba1 immunohistochemistry. AH group demonstrated marked macrophage reduction compared to model mice, whereas AE group exhibited intensified macrophage infiltration (Figure 1E). These findings collectively confirm that healthy gut microbiota transplantation mitigates EMs progression through epithelial remodelling and immune modulation, while EM‐associated microbiota exacerbates disease pathology, establishing gut microbiome's pivotal role in EMs pathogenesis.

### 3.3 The Impact of Human FMT on Gut Microbiota Diversity

**FIGURE 2:**
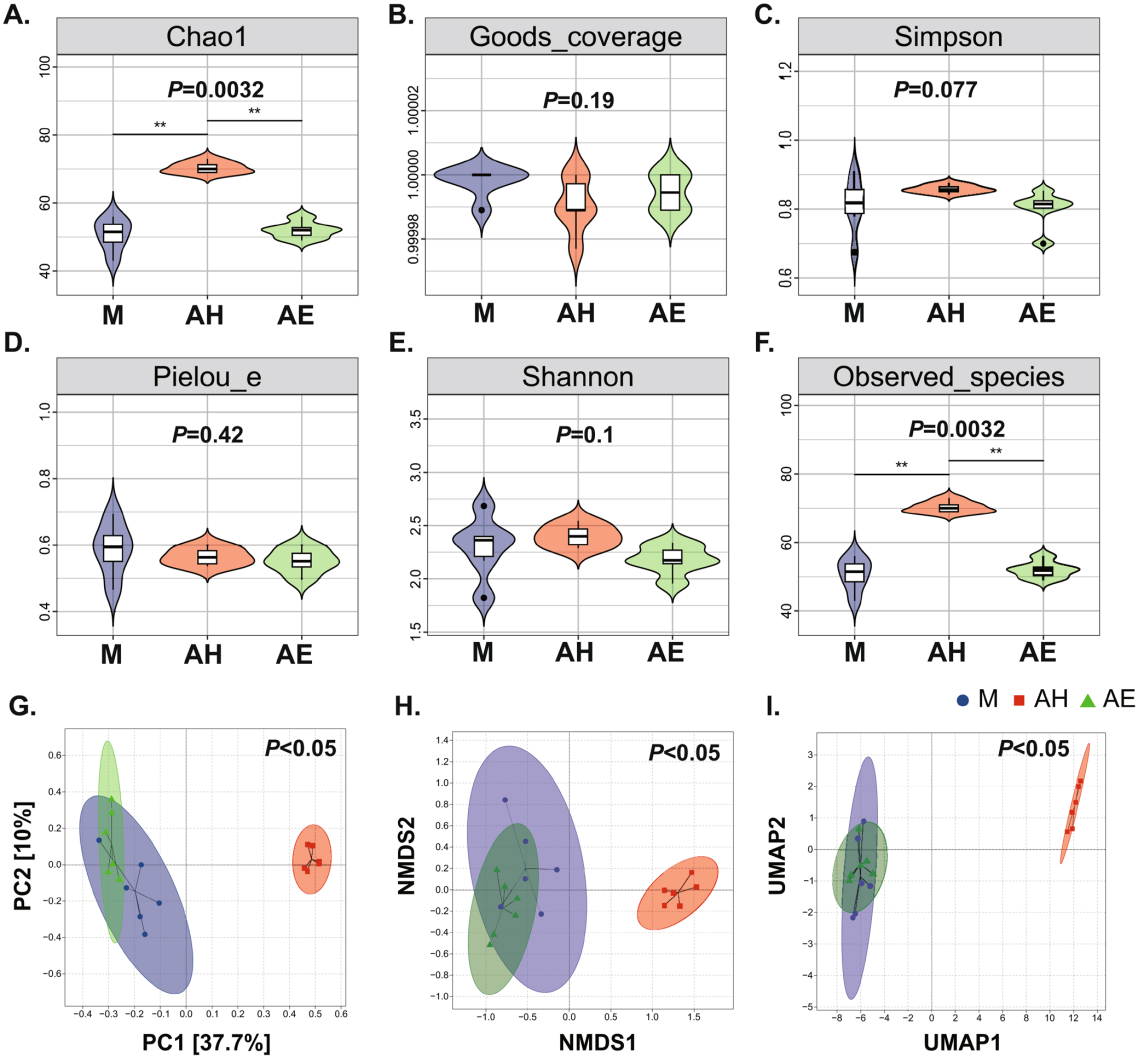
FMT on gut microbiota diversity in a mouse model of endometriosis. (A) Chao1 index. (B) Good's coverage index. (C) Simpson index. (D) Pielou's evenness index. (E) Observed species index. (F) Observed_species index. (G) Principal coordinate analysis (PCoA). (H) Non‐metric multidimensional scaling (NMDS). (I) Uniform Manifold Approximation and Projection (UMAP) analysis. AE, FMT from endometriosis patients; AH, FMT from healthy women; M, model group. Data are presented as mean ± standard deviation. *p < 0.05, **p < 0.01, ***p < 0.001, ****p < 0.0001.

To explore the role of intestinal microbial communities in EMs pathogenesis, faecal specimens from recipient mice underwent 16S ribosomal RNA sequencing. This approach enabled analysis of taxonomic shifts and α‐diversity alterations after EMs induction and FMT administration. Results from rarefaction curves and species accumulation curves indicated that the sequencing volume and depth were sufficient (Figure S1A,B). Analysis of α‐diversity (Figure 2A–F) revealed that the AH group exhibited significantly higher α‐diversity indices, including Chao1 index and observed species, compared to the M group (*p* < 0.01), with the AH group also showing significantly greater diversity than the AE group (*p* < 0.01). These findings suggest enhanced microbial richness in the AH group. Principal coordinate analysis (PCoA) further validated alterations in bacterial diversity, with distinct clustering patterns observed among the AH, M and AE groups in the PCoA plot, indicating significant differences in gut microbial community structure (*p* < 0.05). Notably, the AH group demonstrated significant separation from the M and AE groups along the PC1 axis, highlighting its unique gut microbiota profile compared to the other groups (Figure 2G). As shown in Figure 2H, non‐metric multidimensional scaling (NMDS) analysis also revealed distinct clustering among the AH, M and AE groups, further confirming significant structural differences in gut microbiota (*p* < 0.05). Similarly, Uniform Manifold Approximation and Projection (UMAP) analysis (Figure 2I) reinforced the conclusion of marked divergence in microbial community structure across groups (*p* < 0.05), with the AH group again displaying clear separation from the M and AE groups. Collectively, these data reveal that the healthy donor‐derived microbiota (AH group) maintains superior microbial diversity and ecological stability relative to both endometriosis‐model (M) and patient‐FMT (AE) groups. This divergence aligns with the hallmark features of intestinal eubiosis versus dysbiosis, respectively. The observed microbiota‐host disequilibrium provides mechanistic insights into endometriosis pathogenesis, while microbiota‐targeted interventions may offer novel therapeutic avenues for restoring symbiosis.

### 3.4 The Influence of Human FMT on the Structure of the Gut Microbiota

**FIGURE 3:**
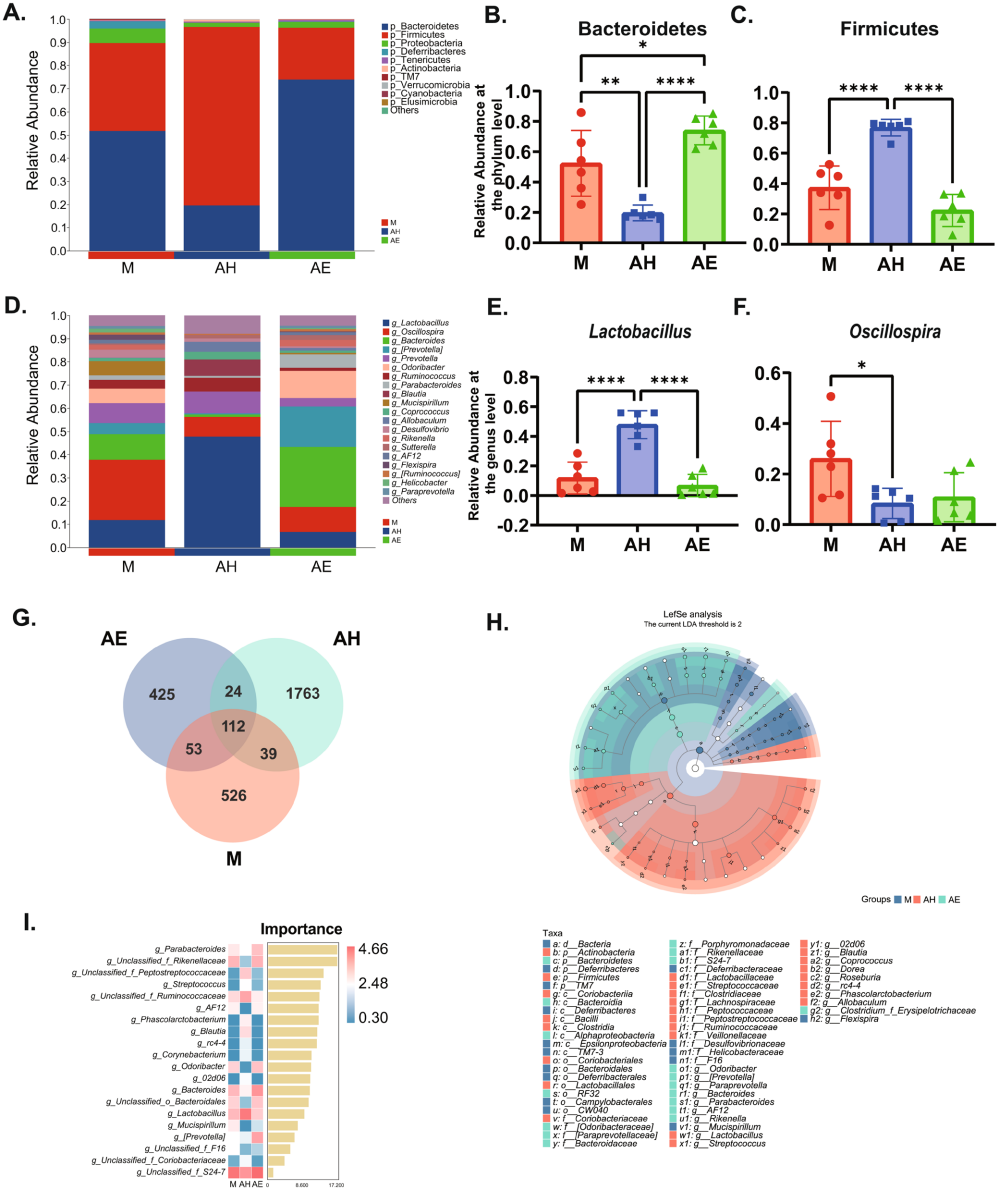
Effects of FMT on gut microbiota composition in a mouse model of endometriosis. (A) Relative abundance of gut microbiota at the phylum level. (B) Relative abundance of Bacteroidetes. (C) Relative abundance of Firmicutes. (D) Relative abundance of gut microbiota at the genus level. (E) Relative abundance of *Lactobacillus*. (F) Relative abundance of *Oscillospira*. (G) Venn diagram of microbial taxa distribution. (H) LEfSe analysis highlighting differentially abundant taxa. (I) Feature importance heatmap of gut microbiota at the genus level. AE, FMT from endometriosis patients; AH: FMT from healthy women; M, model group. Data are presented as mean ± standard deviation. *p < 0.05, **p < 0.01, ***p < 0.001, ****p < 0.0001.

To further elucidate the effects of FMT on gut microbiota composition, a comprehensive taxonomic profiling of the intestinal microbial communities was performed. The analysis revealed that Firmicutes and Bacteroidetes constituted the predominant microbial taxa at the phylum level (Figure 3A). Compared to the M group, the relative abundance of Bacteroidetes was significantly reduced in the AH group (*p* < 0.01), whereas it markedly increased in the AE group (Figure 3B, *p* < 0.05). In contrast, the abundance of Firmicutes was substantially elevated in the AH group (Figure 3C, *p* < 0.00001) but exhibited a decreasing trend in the AE group. At the genus level, Group AH exhibited higher abundances of *Lactobacillus* and Oscillospira (Figure 3D). Figure 3E,F illustrate the relative abundance changes of *Lactobacillus* and *Oscillospira* across experimental groups, respectively. Compared to the M group, the AH group exhibited a significantly higher abundance of *Lactobacillus* (Figure 3E, *p* < 0.00001), while a declining trend was observed in the AE group. Meanwhile, the abundance of Oscillospira was significantly lower in Group AH than in Group M. Venn diagram analysis revealed the distribution of shared and unique operational taxonomic units (OTUs) among the groups. Group AH possessed the highest number of unique OTUs (1763), while the largest number of shared OTUs (425) was observed between the M group and AE group (Figure 3G). LefSe analysis further identified significantly different microbial taxa among the groups (Figure 3H). Random forest analysis indicated that Bacteroidetes had the highest importance in the AE group, while Firmicutes was most prominent in the AH group (Figure 3I). These differences may be closely associated with the pathogenesis of endometriosis, indicating that the gut microbiota could play a critical role in the development of this disease.

### 3.5 Healthy Gut Microbiota Transplantation Improves Intestinal Barrier Function by Upregulating Tight Junction Proteins

**FIGURE 4:**
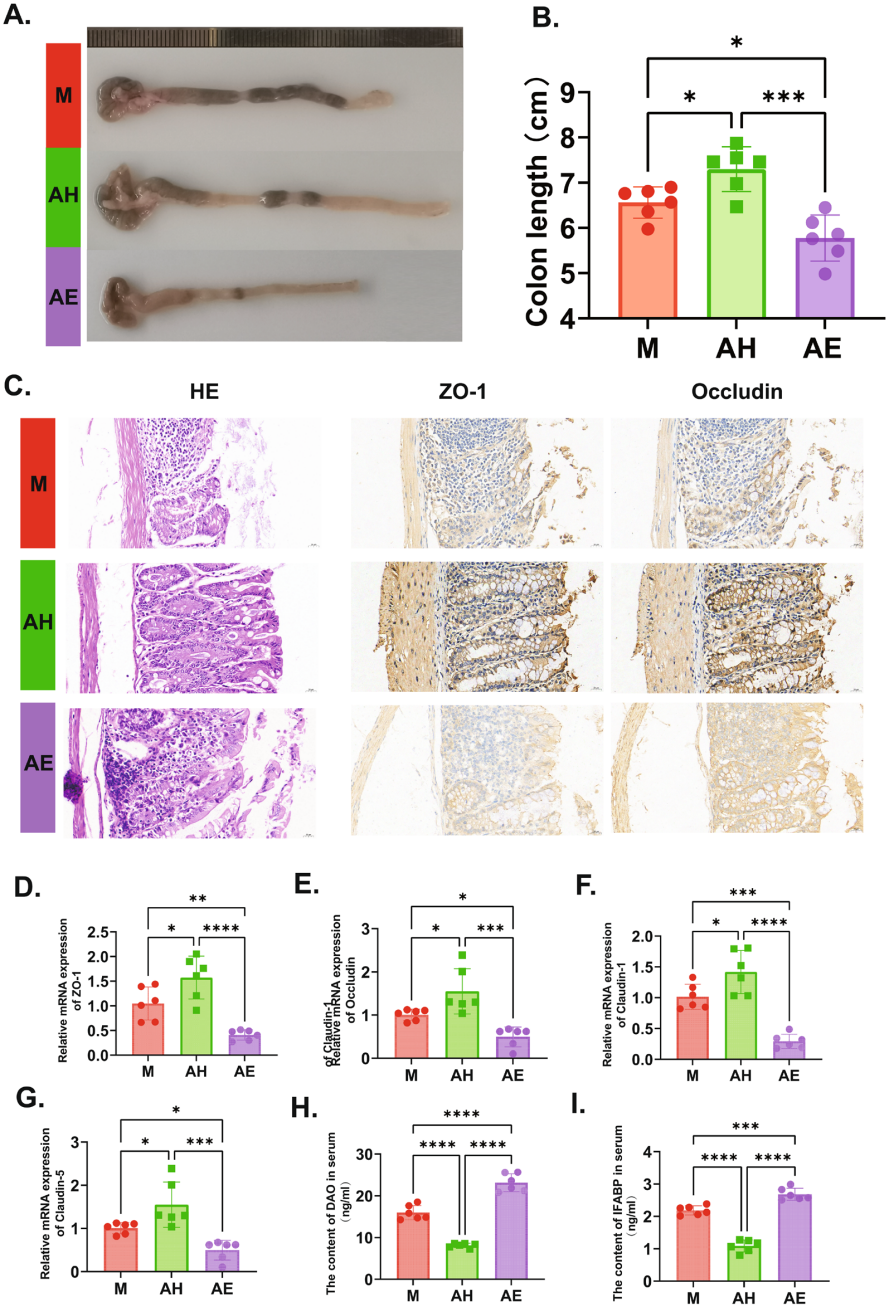
Healthy gut microbiota transplantation improves intestinal barrier function by upregulating tight junction proteins. (A) Representative photographs of colons from each group. (B) Colon length across groups. (C) Histological examination of colonic tissues via H&E staining (left) and immunohistochemical staining for ZO—1 (middle) and Occludin (right), with a scale bar of 50 μm under 20× magnification; Relative mRNA expression levels of tight junction—associated genes in colonic tissues: ZO—1 (D), Occludin (E) Claudin—1 (F), Claudin—5 (G). (H, I) Concentrations of DAO (H) and IFABP (I) in serum, indicators of intestinal mucosal injury. Scale bar: 20 μm. AE, FMT from endometriosis patients; AH, FMT from healthy women; M, model group. Data are presented as mean ± standard deviation. *p < 0.05, **p < 0.01, ***p < 0.001, ****p < 0.0001.

To investigate the effects of FMT on intestinal inflammation and barrier function, colonic specimens were collected from all experimental groups for morphometric and histopathological analyses. Comparative assessment revealed that the AH group exhibited significantly longer colon length than the M group (7.298 ± 0.4959 vs. 6.562 ± 0.3469 cm, *p* = 0.0002), while the AE group showed significant shortening (5.777 ± 0.5084 cm) (Figure 4A,B). Histological evaluation via haematoxylin–eosin (HE) staining demonstrated distinct pathological features: both M and AE groups displayed typical mucosal degeneration characterised by lamina propria thinning, structural disruption of the mucosal layer, disorganised intestinal villi with focal exfoliation and reduced crypt numbers. In contrast, the AH group maintained intact tissue architecture with orderly epithelial cell arrangement and tight junctions extending into the lamina propria (Figure 4C). Immunohistochemical analysis further indicated that compared with the M group, the AH group exhibited increased expression of intestinal barrier proteins ZO‐1 and Occludin, whereas the AE group showed reduced expression (Figure 4C). To precisely quantify key protein expression levels, RT‐PCR analysis was performed for ZO‐1, Occludin and tight junction proteins Claudin‐1 and Claudin‐5. Results demonstrated that relative to the M group, the AH group displayed significant upregulation of ZO‐1, Occludin, Claudin‐1 and Claudin‐5 (Figure 4D–G, *p* < 0.05), while the AE group exhibited significant downregulation (Figure 4D–G, *p* < 0.01). Given that diamine oxidase (DAO) and intestinal fatty acid‐binding protein (IFABP) are sensitive and specific biochemical indicators for intestinal mucosal injury, serum levels of DAO and IFABP were measured to evaluate the impact of FMT on intestinal permeability. Results showed that compared with the M group, the AH group had significantly elevated serum DAO and IFABP levels, whereas the AE group showed significant reductions (Figure 4H,I, *p* < 0.05). Collectively, these findings demonstrate that gut microbiota profoundly modulates intestinal inflammation and barrier function. Specifically, transplantation of gut microbiota from healthy donors (AH group) effectively alleviated intestinal mucosal damage and permeability abnormalities by maintaining structural integrity of colonic tissues and upregulating expression of intestinal barrier proteins (ZO‐1, Occludin, Claudin‐1 and Claudin‐5).

### 3.6 Faecal Microbiota Transplantation From Healthy Donors Specifically Elevates Acetate Levels in Faeces and Ectopic Lesions of Mice

**FIGURE 5:**
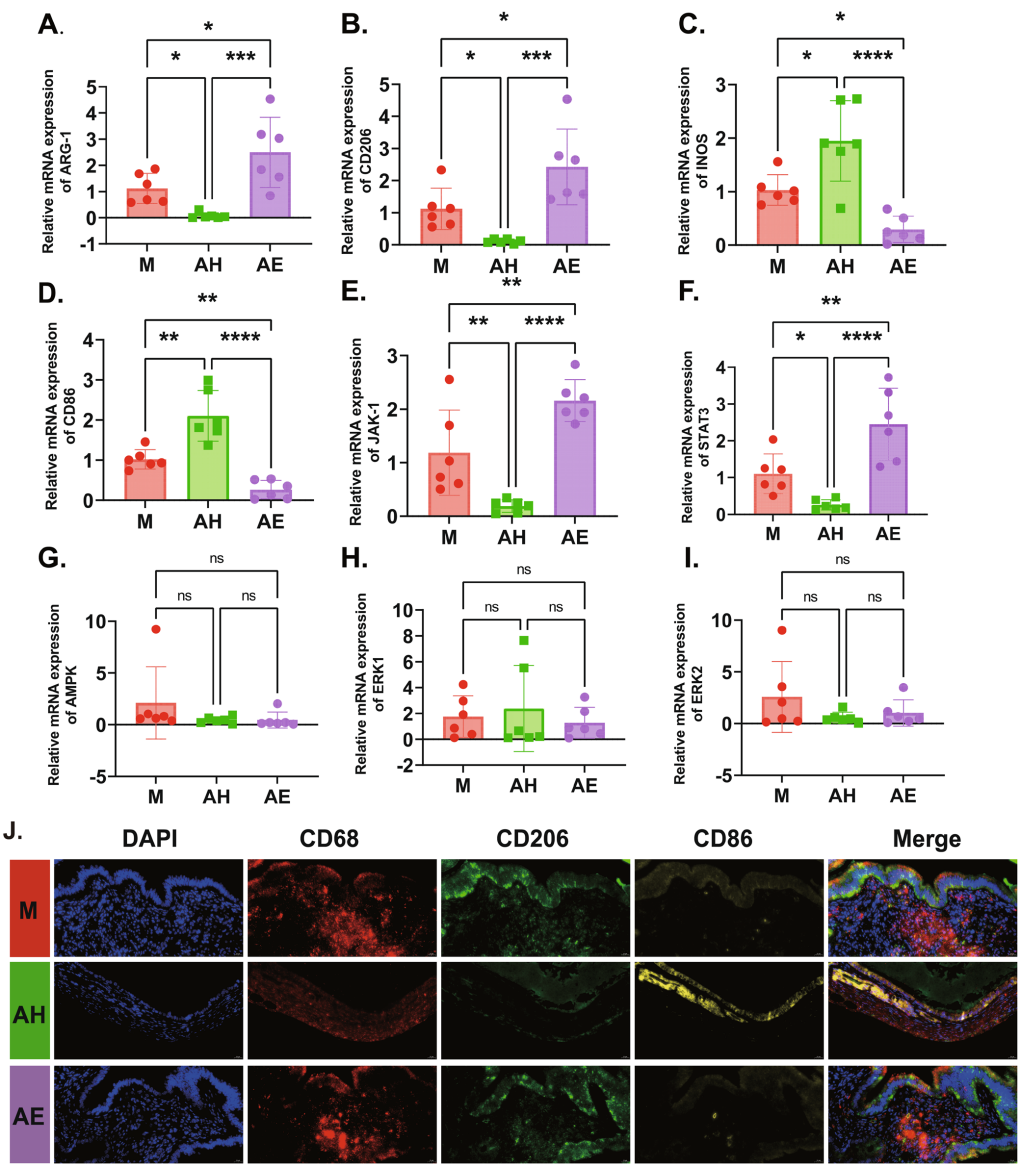
Healthy Donor FMT Promotes M1 Macrophage Polarisation via JAK‐1/Stat3 Signalling Axis in Endometriosis Lesions. (A–D) Relative mRNA expression levels of macrophage polarisation—associated marker genes in colonic tissues: ARG—1 (A), CD206 (B), iNOS (C), and CD86 (D). (E–I) Relative mRNA expression levels of genes involved in pathways regulating macrophage polarisation in Endometriotic tissues: JAK1 (E), STAT3 (F), AMPK (G), ERK1 (H), ERK2 (I). (J) Immunofluorescence staining of colonic tissues for macrophage markers: DAPI for nuclei, CD68 for total macrophages, CD206 for M2—type macrophages, CD86 for M1—type macrophages, and merged images. Scale bar: 20 μm. AE, FMT from endometriosis patients; AH, FMT from healthy women; M, model group. Data are presented as mean ± standard deviation. *p < 0.05, **p < 0.01, ***p < 0.001, ****p < 0.0001.

To investigate the mechanism by which faecal microbiota transplantation (FMT) affects the progression of EMs, immunofluorescence staining and qPCR were used to detect macrophage polarisation markers and related signalling pathways in ectopic lesions. qPCR results (Figure 5A–D) showed that compared with the model group (M), the mRNA expression levels of M1 macrophage markers iNOS (Figure 5C) and CD86 (Figure 5D) in the healthy donor FMT group (AH) were significantly increased (*p* < 0.01), while the mRNA expression levels of M2 markers Arg1 (Figure 5A) and CD206 (Figure 5B) were significantly decreased (*p* < 0.05). In contrast, the mRNA expression levels of iNOS and CD86 in the AE group were significantly decreased (*p* < 0.01), and the mRNA expression levels of M2 markers Arg1 (Figure 5A) and CD206 (Figure 5B) were significantly increased (*p* < 0.01). Immunofluorescence double staining (Figure 5J) showed that the co‐localisation signal of CD86^+^/CD68^+^ (M1 macrophages) cells in the AH group was stronger than that in the M group, while the co‐localisation signal of CD206^+^/CD68^+^ (M2 macrophages) cells was weaker than that in the M group. The co‐localisation signals of the two types of cells in the AE group were similar to those in the M group. To further explore the molecular mechanism, qPCR was used to detect common signalling pathways involved in macrophage polarisation. The results showed that compared with the model group (M), the mRNA expressions of JAK‐1 and STAT3 (Figure 5E,F) in the AH group were significantly upregulated (*p* < 0.01), indicating activation of the JAK‐1/Stat3 signalling axis, while the mRNA expression levels of AMPK, ERK1 and ERK2 showed no significant changes (Figure 5G–I). These results indicate that FMT from healthy donors may promote macrophage polarisation toward the M1 phenotype and inhibit M2 polarisation by activating the JAK‐1/Stat3 signalling axis, thereby regulating the immune microenvironment of EMs lesions.

### 3.7 Faecal Microbiota Transplantation From Healthy Donors Specifically Elevates Acetate Levels in Faeces and Ectopic Lesions of Mice

**FIGURE 6:**
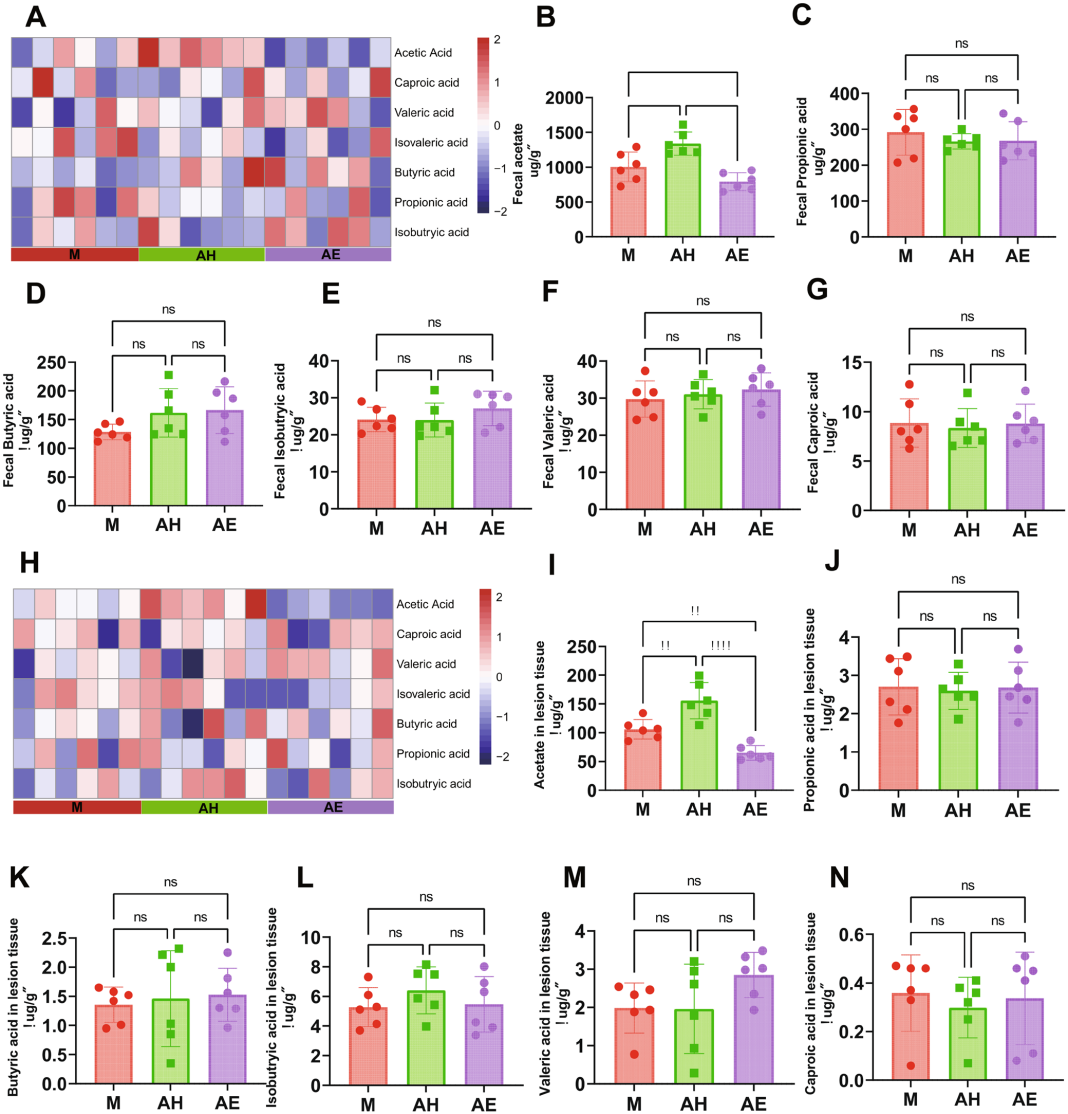
Faecal microbiota transplantation from healthy donors specifically elevates acetate levels in faeces and ectopic lesions of mice. (A) Heatmap of SCFA relative levels in faeces among groups. (B–G) Quantification of faecal SCFAs: Propionic acid (B), Butyric acid (C), Isobutyric acid (D), Valeric acid (E), Caproic acid (F), Isovaleric acid (G). (H) Heatmap of SCFA relative levels in endometriotic lesions among groups. (I–N) Quantification of SCFAs in endometriotic lesions: Acetic acid (I), Propionic acid (J), Butyric acid (K), Isobutyric acid (L), Valeric acid (M), Caproic acid (N). AE, FMT from endometriosis patients; AH, FMT from healthy women; M, model group. Data are presented as mean ± standard deviation. *p < 0.05, **p < 0.01, ***p < 0.001, ****p < 0.0001.

To investigate the impact of FMT on the intestinal metabolome of mice, this study conducted targeted metabolomics analysis on faeces and ectopic lesions from each group of mice to measure short‐chain fatty acid (SCFA) levels. Faecal metabolomics results showed that compared to the model group (M), the faecal acetate level in the healthy donor FMT group (AH) was significantly increased (1004 ± 86.99 vs. 1339 ± 67.89 μg/g, *p* < 0.0002; Figure 6A,B), whereas the acetate level in the endometriosis (EMs) patient FMT group (AE) was significantly decreased (1004 ± 86.99 vs. 792.2 ± 51.76 μg/g, *p* < 0.01; Figure 6A,B). No significant differences were observed in the levels of faecal propionic acid, butyrate, isobutyric acid, valeric acid or caproic acid among the groups (Figure 6C–G). In lesion tissues, the acetate concentration in the AH group was also significantly higher than in the M group (105.9 ± 6.978 vs. 155.8 ± 12.89, *p* < 0.01; Figure 6H,I), while the AE group was significantly lower than the M group (105.9 ± 6.978 vs. 64.94 ± 5.191, *p* < 0.01; Figure 6H,I). No significant differences were found in the levels of propionic acid, butyrate, isobutyric acid, valeric acid or caproic acid in the lesion tissues among the groups. These results indicate that faecal microbiota transplantation specifically modulates acetate levels in both faeces and ectopic lesions of mice.

### 3.8 Healthy Donor FMT Ameliorates Endometriosis by Promoting M1 Macrophage Polarisation via JAK1/STAT3 Pathway Activation

**FIGURE 7:**
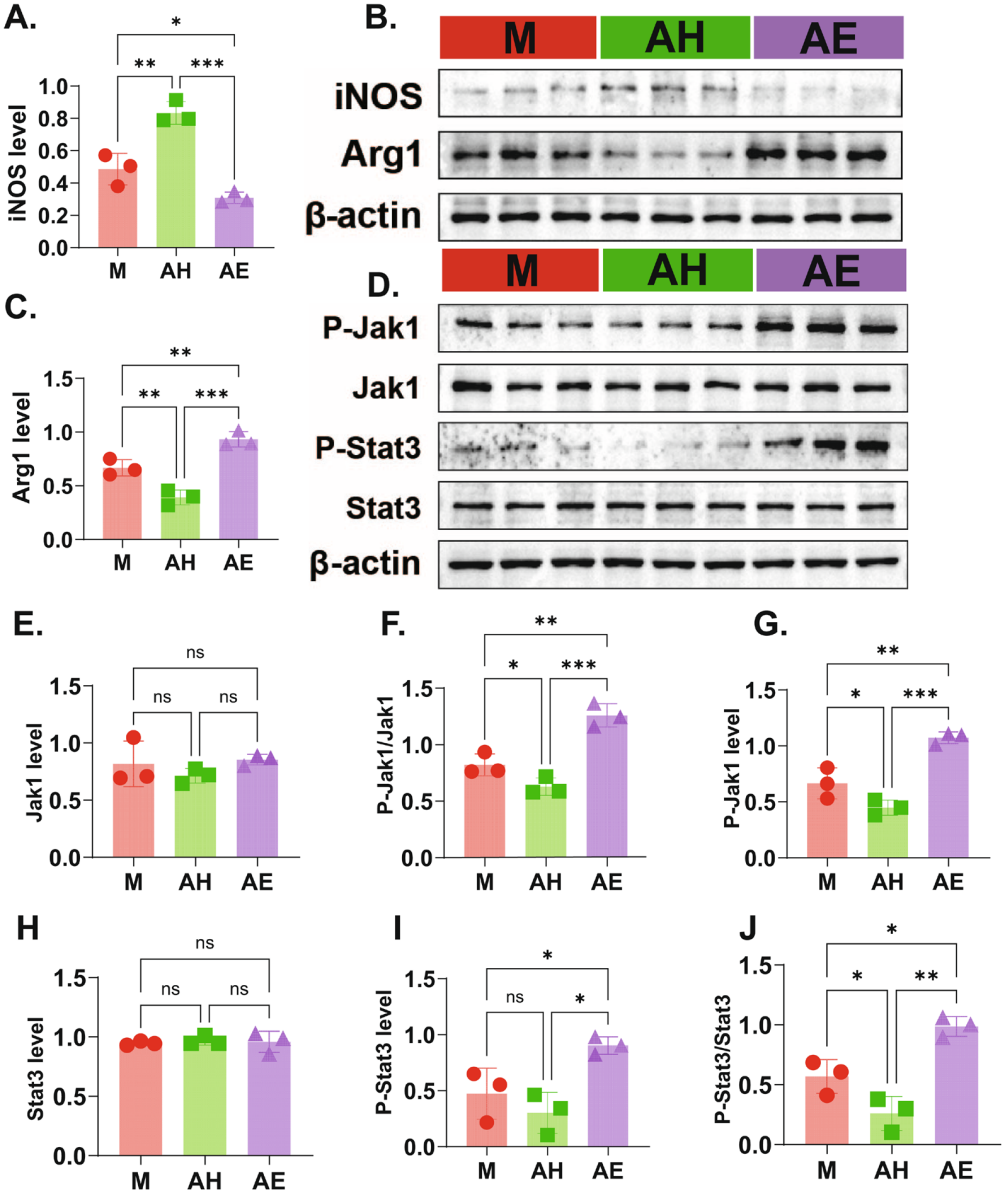
Healthy donor FMT ameliorates endometriosis by promoting M1 macrophage polarisation via JAK1/STAT3 pathway activation. (A, C) Relative protein levels of iNOS (A) and Arg1 (C) in endometriotic tissues, detected by Western blotting. (B) Representative Western blot images of iNOS, Arg1, and β—actin in endometriosis tissues. (D) Representative Western blot images of P—Jak1, Jak1, P—Stat3, Stat3, And β—Actin in endometriosis tissues. (E–J) Quantitative analysis of protein levels: Jak1 (E), P—Jak1/Jak1 Ratio (F), P—Jak1 (G), Stat3 (H), P—Stat3/Stat3 Ratio (I), P—Stat3 (J). AE, FMT from endometriosis patients; AH, FMT from healthy women; M, model group. Data are presented as mean ± standard deviation. *p < 0.05, **p < 0.01, ***p < 0.001, ****p < 0.0001.

**FIGURE 8:**
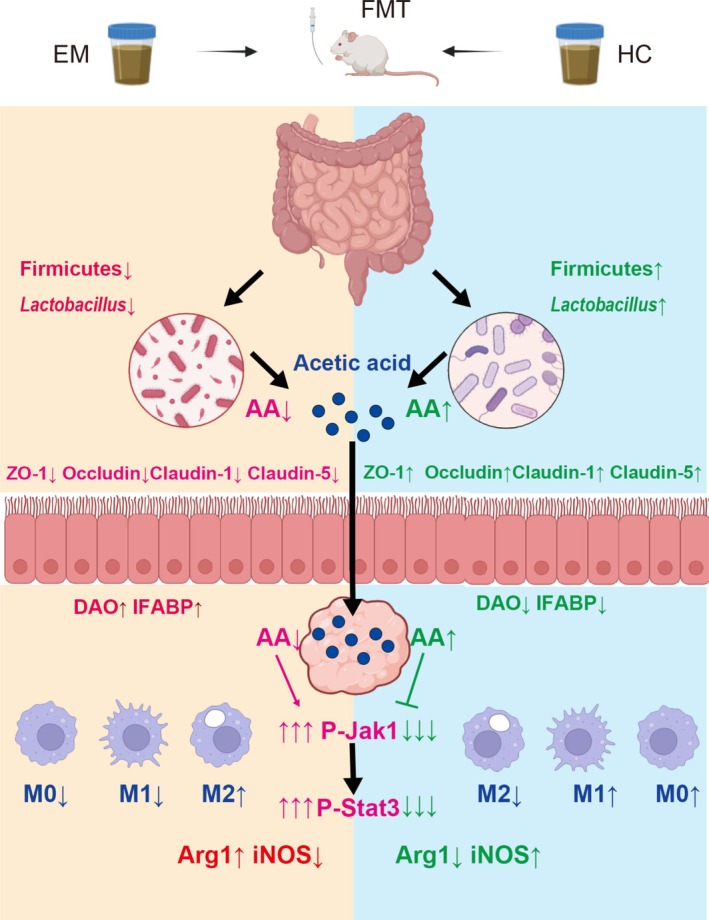
Mechanism of healthy donor faecal microbiota transplantation (FMT) in ameliorating endometriosis via acetate‐mediated JAK1/STAT3 activation and macrophage reprogramming. FMT from healthy donors enriches beneficial gut microbiota (increased Firmicutes, increased *Lactobacillus*), specifically elevating acetate levels in the gut and ectopic lesions. Acetate enhances intestinal barrier integrity by upregulating tight junction proteins (increased ZO‐1, Occludin, Claudin‐1 and Claudin‐5) and reducing intestinal permeability markers (DAO and IFABP). Within lesions, acetate activates the JAK1/STAT3 signalling pathway, driving macrophage polarisation toward the M1 phenotype (increased iNOS and CD86) while suppressing M2 polarisation (decreased Arg1 and CD206), ultimately inhibiting ectopic lesion growth. Conversely, FMT from endometriosis patients exacerbates dysbiosis (decreased Firmicutes and *Lactobacillus*), reduces acetate production, impairs barrier function, fails to activate JAK1/STAT3, and promotes M2‐dominant inflammation, accelerating disease progression.

To investigate the molecular mechanism by which FMT from healthy donors promotes M1 macrophage polarisation, the study examined the expression of macrophage polarisation‐related proteins (iNOS, Arg1) and key proteins of the JAK1/STAT3 signalling pathway in ectopic lesions. The results showed that compared to the model group (M), the expression of iNOS was significantly increased in the healthy donor FMT group (AH) (Figure 7A,B, *p* < 0.01), while the expression of Arg1 was significantly decreased (Figure 7B,C, *p* < 0.01). Conversely, the AE group exhibited the opposite effects. Further analysis of key protein expression in the JAK1/STAT3 signalling pathway within ectopic lesions revealed that the phosphorylation level of JAK1 (p‐JAK1/JAK1 ratio) was significantly elevated in the AH group (Figure 7D,F; *p* < 0.01), and the phosphorylation level of STAT3 (p‐STAT3/STAT3 ratio) was also significantly upregulated (Figure 7D,J,P, *p* < 0.01). However, the total protein levels of JAK1 and STAT3 showed no significant differences among the groups (Figure 7E,H). These findings indicate that healthy donor FMT drives macrophage polarisation toward the M1 phenotype by upregulating the phosphorylation levels of the JAK1/STAT3 signalling pathway, thereby promoting the expression of the M1 macrophage marker iNOS and suppressing the expression of the M2 marker Arg1 (Figure 8).

## 4 Discussion

Our study demonstrates that gut microbiota modulation demonstrates significant potential in the management of endometriosis, particularly through its underlying mechanisms of regulating host immunity and inflammatory responses via microbial metabolites. Although existing studies have indicated the critical role of gut microbiota in the pathogenesis of EMs, the specific mechanisms remain unclear. Consequently, exploring novel therapeutic targets is essential for developing innovative treatment strategies for EMs. This study confirms that gut microbiota significantly ameliorates the pathological progression of EMs by promoting macrophage polarisation toward the M1 phenotype and, for the first time, proposes that the gut microbiota metabolite acetate may serve as a new target for regulating M1 macrophage polarisation to improve endometriosis. This finding not only provides a new perspective for understanding the complex relationship between gut microbiota and EMs but also offers a crucial theoretical foundation for developing novel therapeutic strategies based on gut microbiota metabolites.

Despite existing evidence indicating dysbiosis of the gut microbiota in patients with EMs, the direct causal relationship between gut microbiota and EMs remains unclear (Qin et al. 2022). FMT, a technique that modulates gut microbiota by transplanting faecal bacteria from healthy donors, has emerged as a novel therapeutic approach for treating gut dysbiosis‐related diseases (Ianiro et al. 2022). In recent years, FMT has demonstrated potential in gynaecological conditions such as bacterial vaginosis and polycystic ovary syndrome, yet its effects on EMs have not been investigated (Fenollar and Raoult 2016; Quaranta et al. 2019; Huang et al. 2024). In this study, faecal samples from both EMs patients and healthy women were transplanted into EMs model mice. The results revealed that gut microbiota derived from healthy donors significantly inhibited the growth of EMs lesions, reduced the volume and weight of ectopic lesions (Figure 1B–D), suppressed intestinal inflammatory responses, and enhanced intestinal barrier function (Figure 3). Conversely, gut microbiota from EMs patients exacerbated disease progression. These findings align with previous studies. For instance, Chadchan et al. demonstrated that gut microbiota dysbiosis leads to a reduction in SCFAs, particularly *n*‐butyrate, which inhibits EMs progression by activating G protein‐coupled receptors (GPRs) and suppressing histone deacetylases (HDACs) (Chadchan et al. 2021). Additionally, Wei et al. reported that gut microbiota dysbiosis generates β‐glucuronidase (GUSB), which promotes lesion growth and inflammatory responses by affecting macrophage function (Wei et al. 2023). In this study, FMT‐mediated modulation of gut microbiota significantly increased faecal acetate levels, which may represent a key mechanism for inhibiting EMs progression through the regulation of macrophage polarisation.

Regarding the long‐term stability of FMT‐modulated microbiota, current evidence suggests that engraftment success varies across diseases and donor‐recipient compatibility (Ianiro et al. 2022). While our study observed sustained effects at 4 weeks post‐FMT, longitudinal human studies indicate that microbiota remodelling can persist for months in conditions like inflammatory bowel disease after a single FMT (Ianiro et al. 2022). Future work should track long‐term microbiota stability and correlate it with acetate levels and clinical outcomes in chronic EMs models.

Macrophage polarisation plays a crucial role in the pathogenesis and progression of EMs by regulating inflammatory responses and immune microenvironments. To further investigate the mechanism by which the gut microbiota ameliorates EMs, we analysed the infiltration levels of M1‐type (CD86^+^), M2‐type (CD206^+^), and total (CD68^+^) macrophages in ectopic lesions across different mouse groups using immunofluorescence staining. The results revealed that the mRNA and protein expression of M1 markers (iNOS and CD86) significantly increased in ectopic lesions of the AH group (FMT from healthy donors), whereas M2 markers (Arg1 and CD206) markedly decreased (Figure 5A–DJ). Immunofluorescence double staining demonstrated enhanced co‐localisation signals of CD86^+^/CD68^+^ (M1) cells and weakened signals of CD206^+^/CD68^+^ (M2) cells, suggesting that the healthy microbiota remodels the immune microenvironment by promoting M1 polarisation while suppressing M2 polarisation. This paradigm aligns with established evidence implicating macrophages in EMs progression. Hogg et al. (2021) demonstrated that lesion‐resident macrophages originate from peritoneal macrophages and monocytes, and their depletion significantly inhibits lesion growth. This finding resonates with our observation of reduced lesion size and macrophage infiltration in EMs mice receiving healthy FMT (Figure 5). Critically, our study extends this understanding by revealing that healthy FMT not only reduces total macrophage infiltration but also significantly increases the M1/M2 ratio within lesions. This suggests gut microbiota modulation specifically drives macrophage polarisation toward the classically activated M1 phenotype, a shift associated with attenuated EMs progression. Supporting the therapeutic potential of modulating macrophage polarisation, Yan et al. (2023) demonstrated that intraperitoneal heat‐killed *E. coli* suppresses EMs lesion growth via IL‐1 signalling and M1 polarisation of bone marrow‐derived macrophages. Complementarily, Li et al. (2021) showed that M1‐like macrophage‐derived nanovesicles (M1NVs) reprogram M2‐like macrophages toward the M1 phenotype, effectively inhibiting the migratory and invasive capacities of EM‐derived stromal cells. Conversely, Miller et al. (2020) provided compelling evidence using in vitro and in vivo models that IL‐17A promotes macrophage recruitment and M2 polarisation, enhancing angiogenesis and fueling lesion growth. These contrasting roles underscore a critical balance: M2‐polarised macrophages facilitate lesion development, whereas M1‐polarised macrophages exert inhibitory effects, potentially through pro‐inflammatory cytokine release.

To further investigate the specific mechanisms by which FMT promotes macrophage polarisation, this study conducted 16S rDNA sequencing to further analyse changes in gut microbiota. The results revealed that EMs model mice exhibited significant gut microbiota dysbiosis, with the gut microbiota of the healthy donor group showing significantly better α‐diversity and β‐diversity compared to the model group and the EMs patient group (Figure 2). The M group was dominated by Firmicutes and Bacteroidetes; in the AH group, Firmicutes increased, Bacteroidetes decreased and the *Lactobacillus* genus increased; whereas the AE group showed increased Bacteroidetes and decreased Firmicutes, which was highly consistent with Chadchan's findings (Figure 3). The changes in gut microbiota of EM mice are closely associated with intestinal barrier damage, specifically manifested as disruption of colonic tissue structure, increased infiltration of inflammatory cells, significant downregulation of intestinal barrier‐related proteins (ZO‐1, Occludin, Claudin‐1/5) and elevation of intestinal permeability markers (DAO, IFABP) (Figure 4). This is consistent with Ni's research, which revealed that EMs model mice receiving FMT from EMs mice showed reduced expression of ZO‐2 protein in the intestinal wall, while intraperitoneal lipopolysaccharide (LPS) levels were significantly elevated. Exogenous supplementation with α‐linolenic acid improved the gut microbiota, intestinal barrier and intra‐abdominal inflammatory environment in EMs mice and reduced LPS levels (Ni et al. 2021). In summary, FMT reconstructs the gut microbiota to enhance intestinal barrier function, interrupting the vicious cycle in which gut‐derived inflammation drives the progression of ectopic lesions.

In our metabolomic analysis (Figure 6), we identified a striking specificity whereby FMT from healthy donors selectively elevated acetate levels in both faecal samples and ectopic lesions, while other SCFAs remained unchanged. This acetate dominance, driven by enriched Lactobacillus (Figure 3E), was absent in AE‐FMT mice, where acetate levels significantly declined. The accumulation of acetate within lesions suggests its direct role in modulating the ectopic microenvironment, positioning it as the pivotal microbial metabolite orchestrating anti‐EMs effects. Mechanistically, this acetate‐specific increase arises from distinct microbiota remodelling in which FMT enriched acetate‐producing Lactobacillus but did not significantly alter butyrate‐producing taxa, diverging from Chadchan et al.'s observation of depleted butyrogenic bacteria in EMs (Chadchan et al. 2023, 2021). While Chadchan et al. emphasised butyrate's role via GPCR/HDAC pathways, our data establish acetate as the primary effector in FMT‐mediated EMs amelioration. Specifically, acetate drives JAK1/STAT3‐mediated M1 macrophage polarisation to remodel the inflammatory microenvironment, whereas butyrate directly suppresses lesion cells via GPCR/HDAC inhibition (Chadchan et al. 2021). This expands the therapeutic scope of microbiota‐derived metabolites, highlighting acetate's unique role in FMT‐mediated modulation of gut microbiota to inhibit EMs progression through immune reprogramming. While acetate emerged as the pivotal metabolite, future studies should assess contributions from bile acids, tryptophan derivatives and other immunomodulatory microbial metabolites.

From a mechanistic perspective, FMT from healthy donors significantly increased the relative abundance of *Lactobacillus* (Figure 3E)—a beneficial genus that maintains gut microbiota balance and enhances intestinal barrier function (Berni 2019). This enrichment drove a specific elevation of acetate levels in both faeces and ectopic lesions, while other SCFAs remained unchanged (Figure 6). Crucially, this acetate accumulation directly activated the JAK1/STAT3 pathway within lesions (Figure 7), evidenced by upregulated phosphorylation of JAK1 and STAT3 without altering total protein levels. The phosphorylation cascade correlated with iNOS upregulation and Arg1 suppression, confirming acetate‐driven M1 polarisation. Notably, AMPK and ERK1/2 pathways were unaffected (Figure 5G–I), highlighting the selectivity of JAK1/STAT3 activation. This aligns with *Bacteroides*‐derived acetate activating JAK1/STAT3 to promote M1 polarisation in hepatocellular carcinoma, indicating a conserved immunometabolic axis (Machado et al. 2022; Ma et al. 2024; Erny et al. 2021). Research by Ma et al. revealed that acetic acid enhances histone H3K27 acetylation, driving M1‐like macrophage polarisation and enhancing the functionality of cytotoxic CD8^+^ T cells, thereby inhibiting hepatocellular carcinoma (HCC) tumour growth (Ma et al. 2024). In this study, the gut microbiota from healthy donors increased the production of acetate, promoting M1‐like macrophage polarisation and suppressing the progression of EMs. This finding aligns with the role of acetate in regulating macrophage polarisation as reported in the literature. At the mechanistic level, this study revealed that acetate regulates gut barrier function and inflammatory responses, indirectly influencing macrophage polarisation. Nevertheless, the 4‐week intervention window limits evaluation of long‐term therapeutic outcomes such as lesion recurrence and systemic immune safety, necessitating extended observation in future studies.

For future therapeutic translation, combining FMT with low‐dose hormonal therapy may synergistically target both hormonal dependence and inflammation: Hormones suppress ectopic tissue proliferation, while FMT restores immunometabolic homeostasis to prevent recurrence (Horne and Missmer 2022; Berni 2019). This approach could minimise hormonal side effects and enhance efficacy, akin to FMT combined with metformin in polycystic ovary syndrome (Fenollar and Raoult 2016).

While traditional hormonal therapies can temporarily alleviate endometriosis (EMs) symptoms by suppressing oestrogen pathways, their long‐term use is associated with limitations including osteoporosis risk, metabolic disturbances and high recurrence rates after discontinuation (Bedaiwy et al. 2017). In contrast, faecal microbiota transplantation (FMT) remodels the gut ecosystem through non‐hormonal mechanisms, demonstrating multi‐targeted regulatory advantages. FMT restores gut microbiota‐mediated bile acid metabolism (activating FXR/TGR5 signalling) and short‐chain fatty acid (SCFA) production, thereby enhancing mucosal barrier integrity while systemically suppressing pro‐inflammatory cytokines and ectopic lesion activity (Seekatz et al. 2014). Furthermore, FMT circumvents endocrine axis disruption inherent to hormonal therapies, offering a safer alternative for adolescents, perimenopausal patients, and those with metabolic comorbidities (Stuenkel 2021). Notably, animal studies confirm that FMT‐induced microbiota‐immune homeostasis persists for ≥ 12 weeks (Pu et al. 2024), and its demonstrated long‐term efficacy in IBD patients, with microbiota stability lasting up to 24 months or more, supports its therapeutic potential for EMs (Facchin et al. 2024). This sustained modulation may stem from FMT‐derived SCFAs (e.g., butyrate) suppressing inflammation via the GPR43/HDAC‐NF‐κB pathway, promoting Treg differentiation and concurrently improving insulin sensitivity through metabolic targets like PPARγ (Kibbie et al. 2021). Building on these mechanisms, future studies should explore synergistic FMT‐based strategies, such as using low‐dose dienogest (DNG) initially to control lesions rapidly followed by sequential FMT to sustain remission, or co‐administering with prebiotics or specific probiotics to enhance microbial engraftment. Ultimately, personalised microbiota transplantation tailored to patient enterotypes and metabolic phenotypes (Zikou et al. 2024) may establish new paradigms for optimised EMs management.

This study is the first to demonstrate the pivotal role of gut microbiota in the pathogenesis of EMs through FMT. The findings reveal that the gut microbiota from healthy women significantly ameliorates symptoms in EMs mouse models, reduces the volume and weight of ectopic endometrial lesions and enhances intestinal barrier function by modulating the balance of gut microbiota. The potential mechanism involves the promotion of M1 macrophage polarisation through increased intestinal acetate concentration, thereby inhibiting EMs progression. This discovery not only provides novel insights into EMs treatment but also offers new evidence for the role of gut microbiota in immune regulation.

## 5 Conclusion

This study establishes through a murine endometriosis model that faecal microbiota transplantation (FMT) from healthy donors exerts therapeutic effects by remodelling gut microbial composition—specifically enhancing α‐diversity and *Lactobacillus* abundance while suppressing Bacteroidetes—and elevating acetate levels in both the gut and ectopic lesions. Crucially, acetate activates the JAK1/STAT3 signalling pathway within lesions, driving macrophage polarisation toward the M1 phenotype (upregulated iNOS/CD86) and away from the M2 state (downregulated Arg1/CD206). Concomitantly, healthy FMT reinforces intestinal barrier integrity via upregulation of tight junction proteins (ZO‐1, Occludin, Claudin‐1/5) and reduces gut permeability markers (DAO, IFABP). In stark contrast, FMT from endometriosis patients exacerbates disease progression by impairing microbial ecology, diminishing acetate production, failing to activate JAK1/STAT3, promoting M2 polarisation and disrupting gut barrier function. These findings identify acetate as the pivotal microbial metabolite orchestrating macrophage reprogramming and position gut microbiota restoration as a mechanistic foundation for novel therapies against endometriosis.

## Author Contributions

**Yunyun Xu:** writing – original draft, investigation, data curation, validation, resources, formal analysis, visualization. **Yichen Zhu:** methodology, investigation. **Xiaoyun Wu:** software, investigation. **Wan Peng:** investigation, methodology, data curation. **Yanying Zhong:** methodology, investigation. **Yujie Cai:** investigation, data curation. **Wenjing Chen:** investigation, data curation. **Lu Liu:** investigation, data curation. **BuZhen Tan:** writing – review and editing, supervision, conceptualization, funding acquisition. **Tingtao Chen:** project administration, funding acquisition.

## Ethics Statement

The study received review and approval from the Biomedical Research Ethics Committee of the Second Affiliated Hospital of Nanchang University (Approval No. [2024]01), confirming that informed consent has been obtained from all. All procedures related to animal experiments were approved by the Ethics Committee for Animal Welfare of Nanchang University (Approval No: NCULAE‐20221031116) and conducted according to the approved guidelines.

## Consent

The authors have nothing to report.

## Conflicts of Interest

The authors declare no conflicts of interest. The (partial) results of the present study were orally presented in the China Gut Conference 2025 (Ningbo).

## Supporting information


**Data S1:** mbt270202‐sup‐0001‐supinfo.docx.

## Data Availability

The data used to support the findings of this study are available from the corresponding author upon request, and the raw data of 16S rDNA sequencing generated in this study have been deposited under NCBI SRA BioProject numbers PRJNA1274623 (https://www.ncbi.nlm.nih.gov/).
