# Supplementary material for: Gut Microbiota‐Derived Acetate Ameliorates Endometriosis via JAK1/STAT3‐Mediated M1 Macrophage Polarisation
Source: Microb Biotechnol. 2025 Jul 30;18(8):e70202. doi: 10.1111/1751-7915.70202 (PMC12310558; doi:10.1111/1751-7915.70202)
Supplement: Supplementary file 1 — Data S1: mbt270202‐sup‐0001‐supinfo.docx. [file MBT2-18-e70202-s001.docx]

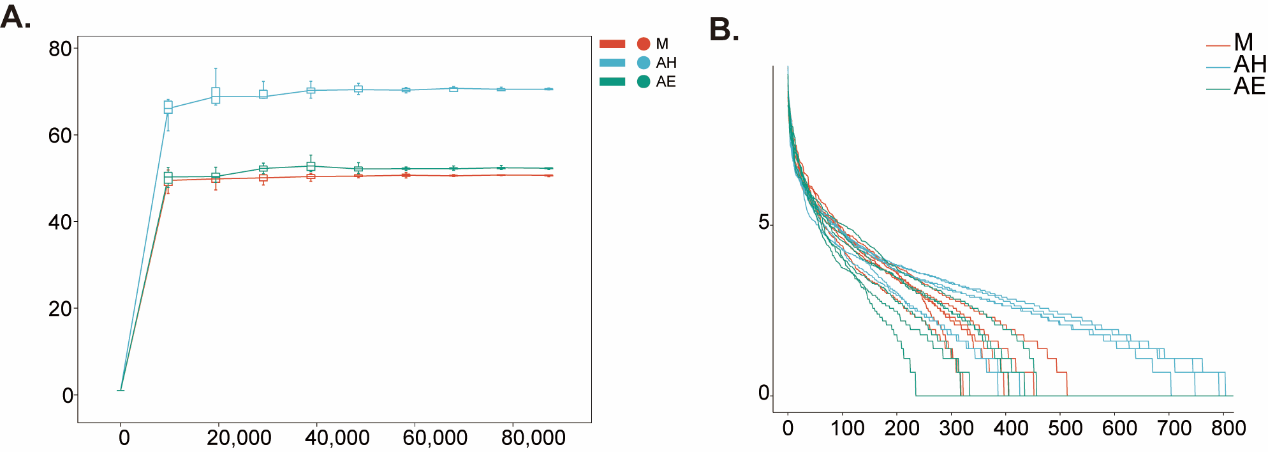
 Supplementary Figure 1. (A) Column chart of the relative abundances of major bacterial genera in different groups;(B) Rarefaction curve.
